# HTLV-1-induced leukotriene B4 secretion promotes the recruitment of target cells

**DOI:** 10.1186/1742-4690-12-S1-P89

**Published:** 2015-08-28

**Authors:** Florent Percher, Patricia Jeannin, Antoine Gessain, Olivier Gout, Pierre-Emmanuel Ceccaldi, Philippe V Afonso

**Affiliations:** 1 Unité EPVO, Institut Pasteur, CNRS UMR 3569, Paris, France; 2 Service de Neurologie, Fondation Ophtalmologique Adolphe de Rothschild, Paris, France; 3 Cellule Pasteur, Université Paris Diderot, Sorbonne Paris Cité, Paris, France

## 

Isolated HTLV-1 viral particles are poorly infectious both in vivo and in vitro. In contrast, the virus is efficiently transmitted through cell-to-cell contact. While the molecular mechanisms of viral cell-to-cell propagation have been extensively studied, the mechanisms facilitating the encounter between an infected cell and a target cell remain unknown. We postulated that the infected cells could behave as a source of chemoattractant, thus recruiting target cells. In 2012, Trindade et al. showed that PBMCs from HTLV-1 infected individuals (HTLV-1 asymptomatic carrier and HAM/TSP patients) could secrete higher levels of leukotriene B4 (LTB4), a potent chemoattractant, than cells from healthy donors. We proposed that, although T cells purportedly secrete no LTB4, LTB4 could be secreted by HTLV-1 infected lymphocytes and facilitate cell-to-cell contacts and viral transmission. We showed by ELISA that HTLV-1 infected lymphocytes (both primary CD4+ T cells and chronically infected cell lines) secrete LTB4. By cell transduction with lentiviral vectors encoding Tax, we suggest that HTLV-1 induced LTB4 secretion is a consequence of Tax-induced cytosolic phospholipase A2 (cPLA2) over expression. Blocking LTB4 secretion (with diverse specific inhibitors of the leukotriene pathway) prevented the recruitment of potential target cells, as well as the formation of cell-to-cell contacts. In conclusion, HTLV-1 diverts the leukotriene pathway and LTB4-mediated chemotaxis facilitates viral propagation.

